# Visual recognition of the anteroposterior female body axis drives spatial elements of male courtship in *Drosophila*

**DOI:** 10.1093/g3journal/jkag037

**Published:** 2026-02-16

**Authors:** Ross M McKinney, Christian Monroy Hernandez, Yehuda Ben-Shahar

**Affiliations:** Department of Biology, Washington University in St. Louis, St. Louis, MO 63130, United States; Department of Biology, Washington University in St. Louis, St. Louis, MO 63130, United States; Department of Biology, Washington University in St. Louis, St. Louis, MO 63130, United States

**Keywords:** *Drosophila melanogaster*, mating behavior, fruit fly, vinegar fly, machine-assisted analysis of behavior

## Abstract

*Drosophila* males exhibit a highly stereotypic courtship ritual toward virgin females, which is comprised of a sequence of specific behavioral elements that depend on inputs from diverse sensory modalities. Particularly, the visual system of the male plays an important role in detecting salient patterns, colors, and motion cues from conspecifics, which can promote or inhibit specific aspects of male courtship such as chase and song production. Here, we use a computer vision and machine learning-based approach, with a simplified courtship paradigm, to show that males also depend on visual cues to determine the anterior–posterior body axis of females, which drives the specific spatial patterns of distinct behavioral courtship elements. We show that the recognition of the female body axis depends, at least in part, on the visual recognition of female eyes as an anterior landmark. Furthermore, we find that in the absence of visual input, courting males adjust not only their relative spatial courtship positioning but also the relative frequencies at which they engage in each specific courtship element. Finally, analyses of the contributions of specific visual projection neurons to the recognition of the female body axis indicate that, although it is driven by a seemingly simple visual cue, the spatiotemporal release patterns of each individual courtship element appear to depend on the activity of multiple independent populations of visual projection neurons. Together, our results provide novel insights into the possible role of visual anatomical features in driving complex social interactions between conspecifics.

## Teaser

This study shows that *Drosophila* males anatomically recognize the anterior-posterior body axis of courted females, which drives the correct spatial release of different behavioral elements via a complex integration of visual cues within the visual system. Therefore, this study should be of general interest to a broad readership because it provides mechanistic insights into a stereotypic, genetically-tractable social behavior, which is relevant to researchers interested in the role of the visual system in driving social interactions, circuit-level integration of mating-related visual cues, and future studies of the genetic and genomic mechanisms that underlie the evolution of mating behaviors and speciation.

## Introduction

Multisensory integration plays a critical role in the courtship ritual of the vinegar fly *Drosophila melanogaster* (Villella and Hall 2008; Krstic et al. 2009; Yamamoto et al. 2014; Rings and Goodwin 2019; Shiozaki et al. 2024). This well studied behavior is characterized by a series of stereotypic innate motor output elements, which are precisely regulated across time and space (Markow 1987; Ning et al. 2022; Fedotov et al. 2026). Previous studies by us and others showed that individual components of the courtship ritual are driven via the integration of visual, auditory, olfactory, gustatory, and mechanosensory cues by dedicated neuronal circuits, which are responsible for the release of context-appropriate behaviors (Ben-Shahar et al. 2010; Lu et al. 2012; Pavlou and Goodwin 2013; Lu et al. 2014; Seeholzer et al. 2018; Leitner and Ben-Shahar 2020; Ning et al. 2022; Vernier et al. 2023). For example, male courtship relies heavily on pheromonal signals detected by chemosensory neurons to identify sex and mating status of their courtship target, while auditory feedback from females in response to the courtship song of males helps modulate song structure and persistence (Ejima and Griffith 2008; Moon et al. 2009; Lu et al. 2012; Thistle et al. 2012; Toda et al. 2012; Vijayan et al. 2014; Clowney et al. 2015; Zhang et al. 2020; Hindmarsh Sten et al. 2021; Baker et al. 2022; Shiozaki et al. 2024; Ding and Lillvis 2025). In addition, visual cues contribute to target tracking and orientation of courting males toward courted females (Willmund and Ewing 1982; Agrawal et al. 2014; Kohatsu and Yamamoto 2015; Ribeiro et al. 2018; Schretter et al. 2025).

**Fig. 1.**
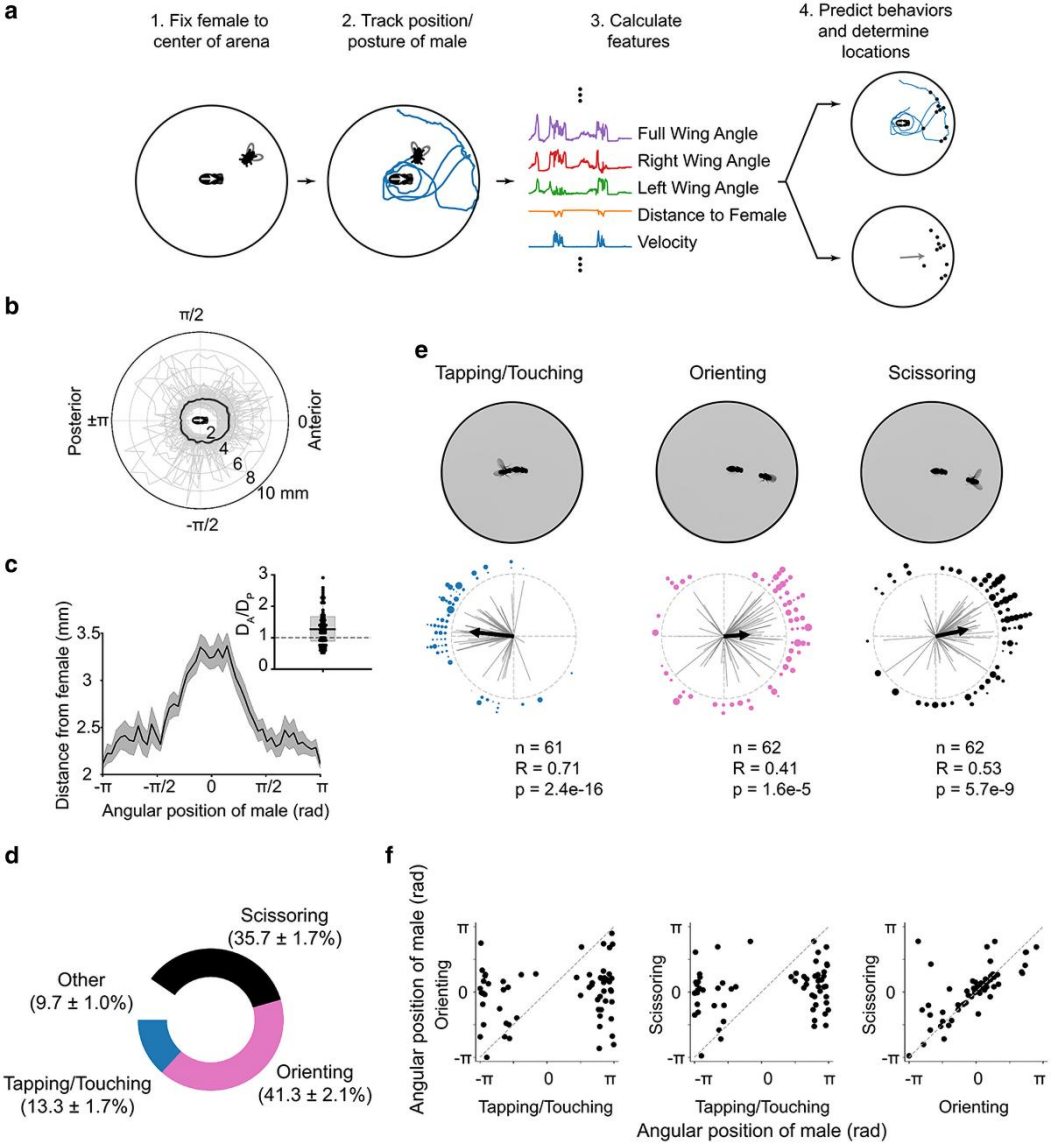
Independent elements of the male courtship ritual follow stereotyped spatiotemporal patterns around the female body axis. a) Overview of algorithm used to determine locations of male mating behaviors during courtship: (i) a female is first fixed to the center of a courtship arena, and then a male is introduced and allowed to court the female for 10 min; (ii) the male is tracked; (iii) tracked features are used in a boosted decision tree classifier to predict frames containing a behavior of interest (ellipses above and below features denote that other features were used in behavioral predictions, see Supplementary Table 2 for full list); (iv) the position of the male in positively classified frames (show by black points) is determined, and the mean angular position over the trial is recorded (shown by gray arrow). b) The average courtship path of Canton-S males (*n* = 62) over the course of a courtship trial. Each thin gray line represents the average path of an individual male. The thick black line represents the mean of all males. Note that for each behavioral trial, all male tracks have been rotated with respect to the female such that the female's anterior–posterior axis is aligned along the horizontal with the anterior end near 0 rad and the posterior end near ±*θ* rad. c) The mean courtship path (same as in b) of Canton-S males, shown in Cartesian coordinates. (Inset) The ratio of the maximum male-female distance when the male is on the anterior half of the female (*D_A_*) to when the male is on the posterior half of the female (*D_P_*). Note that this is significantly greater than 1 (*P <* 0.001, 1-sample *T*-test). d) Tapping/Touching, Orienting, and Scissoring account for ∼90% of the behaviors observed during courtship. Numbers in parentheses represent mean percentages (±SEM) of time that males engaged in each behavior (*n* = 62). e) Examples of individual frames containing positively classified behaviors are shown above mean angular locations, across flies, for each behavior. Each black point represents the mean behavioral location of an individual fly, and the size of the point represents the relative duration of time that the male spent engaging in that behavior. The direction of the black arrow represents the mean behavioral location of all flies and the length of the arrow is proportional to the Rayleigh *R*-value for the total population of flies. Individual gray lines correspond to the Rayleigh *R*-value for individual flies. All distributions are significantly different from uniformity (Rayleigh test), and statistics are shown beneath each plot. f) Scatterplots showing the relationships between angular locations of male courtship behaviors. Each point represents the mean angular location of an individual male engaging in Tapping/Touching, Orienting, or Scissoring. (Left) Tapping/Touching locations are largely anti-correlated with Orienting locations. (Middle) Tapping/Touching locations are largely anti-correlated with Scissoring locations. (Right) Scissoring and Orienting locations are correlated with one another.

Nevertheless, how males use visual cues to regulate the precise spatial patterns of the contextual release of the different component of the courtship ritual remains mostly unknown. To bridge this gap, we developed a simplified machine-assisted courtship analysis paradigm, which enabled us to identify some of the specific visual cues used by males to visually recognize the anteroposterior body axis of courted females, which subsequently drives the probability that a specific male courtship element will be released toward the anterior or posterior half of the body of the courted female (Fig. 1a). Based on the impact of genetic, environmental, and surgical manipulations of male vision and female body morphology on the spatial distribution of different components of male courtship, here we propose a model, which stipulates that males use anatomical landmarks to visually identify the anterior–posterior body axis of courted females, which then drive both the distance and relative locations of specific elements within the courtship ritual. Particularly, males seem to use the eyes of females as the primary landmark that defines the anterior end of their courtship target, which increases the likelihood that singing will occur toward the female's head. Together, these data provide a quantitative empirical approach for machine-assisted analyses of spatial behavioral patterns and support the idea that visual recognition of the anterior–posterior body axis of conspecifics plays a role in the regulation of social interactions in insects.

## Materials and methods

### Fly stocks and husbandry

**Fig. 2.**
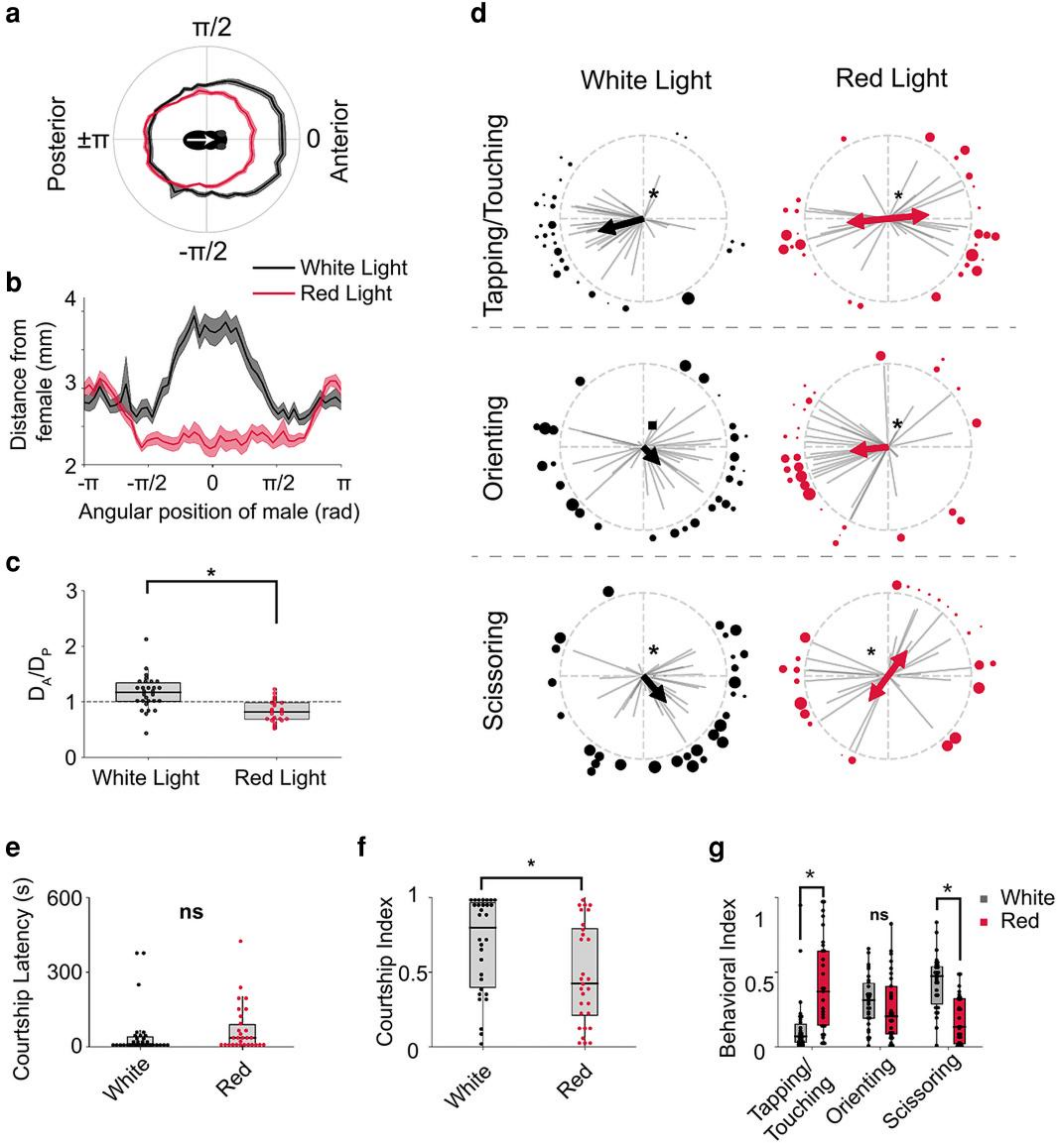
The spatial patterns of stereotyped courtship elements depend on vision. a) Average courtship path for Canton-S males that were allowed to court under either white (black line) or red-light (red line) (*n* = 32/group). b) Same as (a), shown in Cartesian coordinates. c) Maximum distance ratio of male on anterior vs posterior end of female. Males allowed to court under white light had a *D_A_/D_P_ >* 1 (*P <* 0.001, 1-sample *T*-test), whereas males courting under red light had a *D_A_/D_P_ <* 1 (*P <* 0.001, 1-sample *T*-test). d) Average angular positions of males during individual courtship behaviors under either white or red-light. Under white-light, males positioned themselves to the posterior side of the female during bouts of Tapping/Touching (*P <* 10^−5^, Rayleigh Test), whereas they positioned themselves to the anterior side of the female during bouts of either Orienting or Scissoring (Orienting: *P* = 0.08; Scissoring: *P <* 0.01; Rayleigh test). Males courting under red light displayed Tapping and Scissoring behaviors which were bimodally distributed along the anterior–posterior axis of the female; double arrow indicates axis along which bimodality was detected, and the length of each half of arrow is proportional to the distribution's Rayleigh *R*-value (*P <* 0.001 for Tapping/Touching and *P <* 0.05 for Scissoring; Bimodal Rayleigh test). Additionally, males courting under red light Oriented toward the female while on her posterior half (*P <* 0.01; Rayleigh test). e) There was no significant difference in the amount of timed it took males to start courting females under either white or red light (*P >* 0.05; Kruskal test). f) Males courting females under red light courted for shorter durations than males courting females under white light (*P <* 0.01; Kruskal test). g) Behavioral indices are shown for each of the classified behaviors as a fraction of total courtship. Males courting under red light had greater levels of tapping (*P <* 0.001, Kruskal test) and lower levels of Scissoring (*P <* 0.001, Kruskal test) than males courting under white light.

**Fig. 3.**
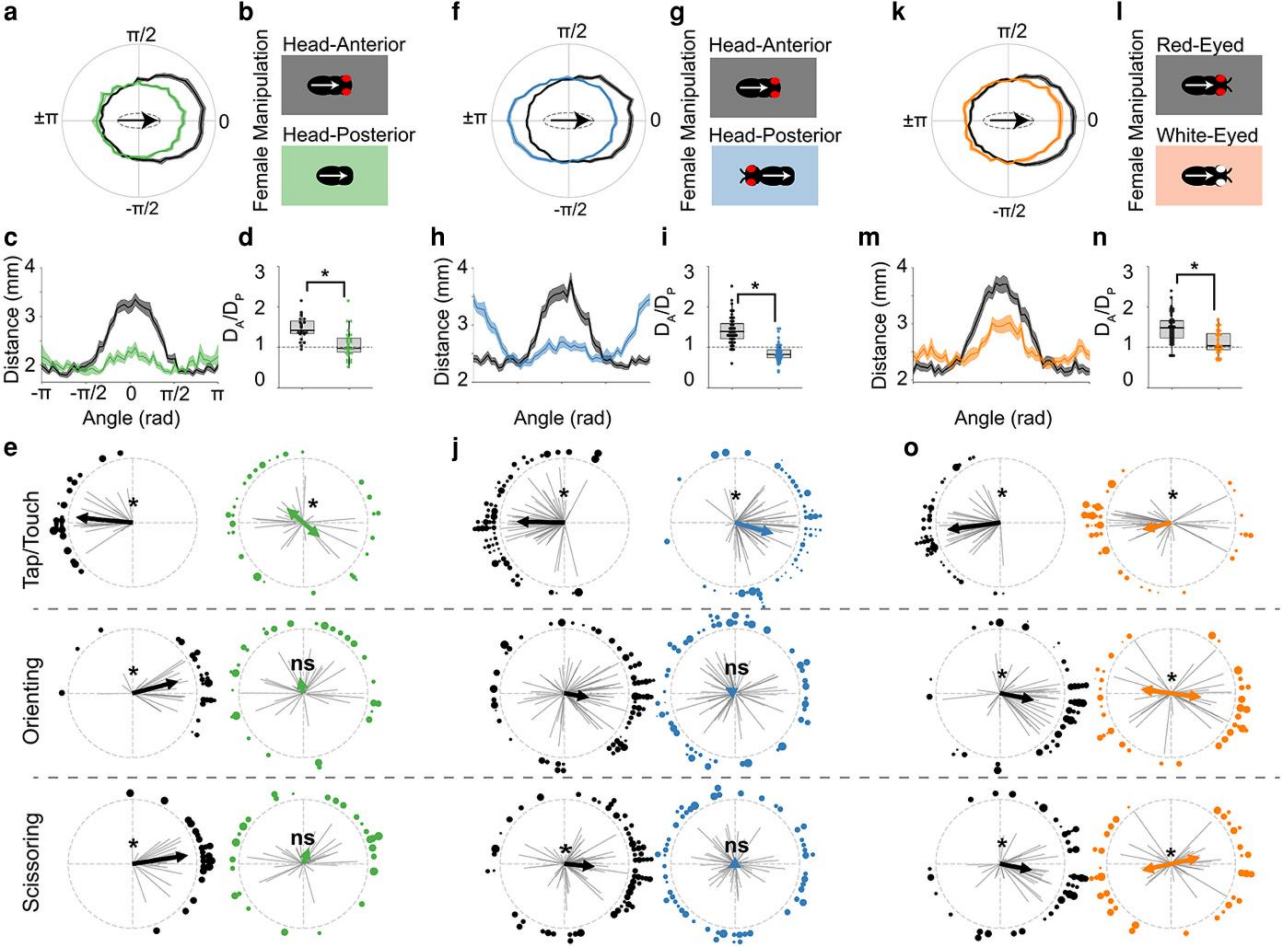
Males use the morphological features of the eyes of females to establish the anterior–posterior body axis of their courtship target. a) Average courtship paths of males courting either intact females (“Head-Intact,” *n* = 32, blackline), or females that had been decapitated (“Head-Decap,” *n* = 32, green line). b) Manipulations for intact and decapitated females. Arrows show the anterior–posterior axis of the female as it has been plotted in (a). c) Average courtship paths of males courting either intact or decapitated females (same as in a), shown in Cartesian coordinates. d) Maximum distance ratio of male on the anterior (*D_A_*) vs posterior (*D_P_*) end of the female. Males courting intact females positioned themselves significantly further from the female's anterior end vs their posterior end (*P <* 10^−5^, 1-sample *T*-test). However, males courting decapitated females did not position themselves significantly further when on either side of the female (*P >* 0.1, 1-sample *T*-test). Thus, males courting intact females had a significantly greater *D_A_/D_P_* ratio than males courting decapitated females (*P <* 10^−4^, one-way ANOVA). e) Behavioral locations of males courting either intact or decapitated females (asterisks denote significance at *P <* 0.05, Rayleigh test or Bimodal Rayleigh test). Note that females are oriented as in (a). f) Average courtship paths of males courting either intact females (“Anterior,” *n* = 64, black line), or females that had their heads transplanted to their posterior end (“Posterior,” *n* = 63, blue line). g) Female manipulations of either the “Anterior” or “Posterior” group. Note that arrows are the same as in (b). h) Average courtship path of males courting either “Anterior” or “Posterior” females (same as in F), shown in Cartesian coordinates. i) *D_A_/D_P_* ratio for males courting either Head-Anterior (“Anterior”) or Head-Posterior (“Posterior”) females. “Anterior” males positioned themselves significantly further from the female's anterior rather than posterior end (*P <* 10^−5^, 1-sample *T*-test), whereas “Posterior” males positioned themselves significantly more distant from the female's posterior rather than anterior end (*P <* 0.01, 1-sample *T*-test). j) Behavioral locations of males courting either “Anterior” or “Posterior” females (asterisks same as in (e). Note that females are oriented as in (f). k) Average courtship path of males courting either females with red eyes (“Red-Eyed”, *n* = 47, black line) or white eyes (“White-Eyed”, *n* = 48, orange line). l) Female manipulations of red-eyed and white-eyed groups. Arrows same as in (b). m) Same as in (k), shown in Cartesian coordinates. n) *D_A_/D_P_* ratio for males courting either red-eyed or white-eyed females. Both groups have ratios significantly different from 1 (red-eyed, *P <* 10^−5^; white-eyed, *P* = 0.03; 1-sample *T*-test); however, males in the Red-Eyed group have significantly greater ratios than males in the white-eyed group (*P <* 10^−5^, one-way ANOVA). o) Behavioral locations of males courting either red-eyed or white-eyed females (asterisks same as in (e). Note that females are oriented as in (k).

**Fig. 4.**
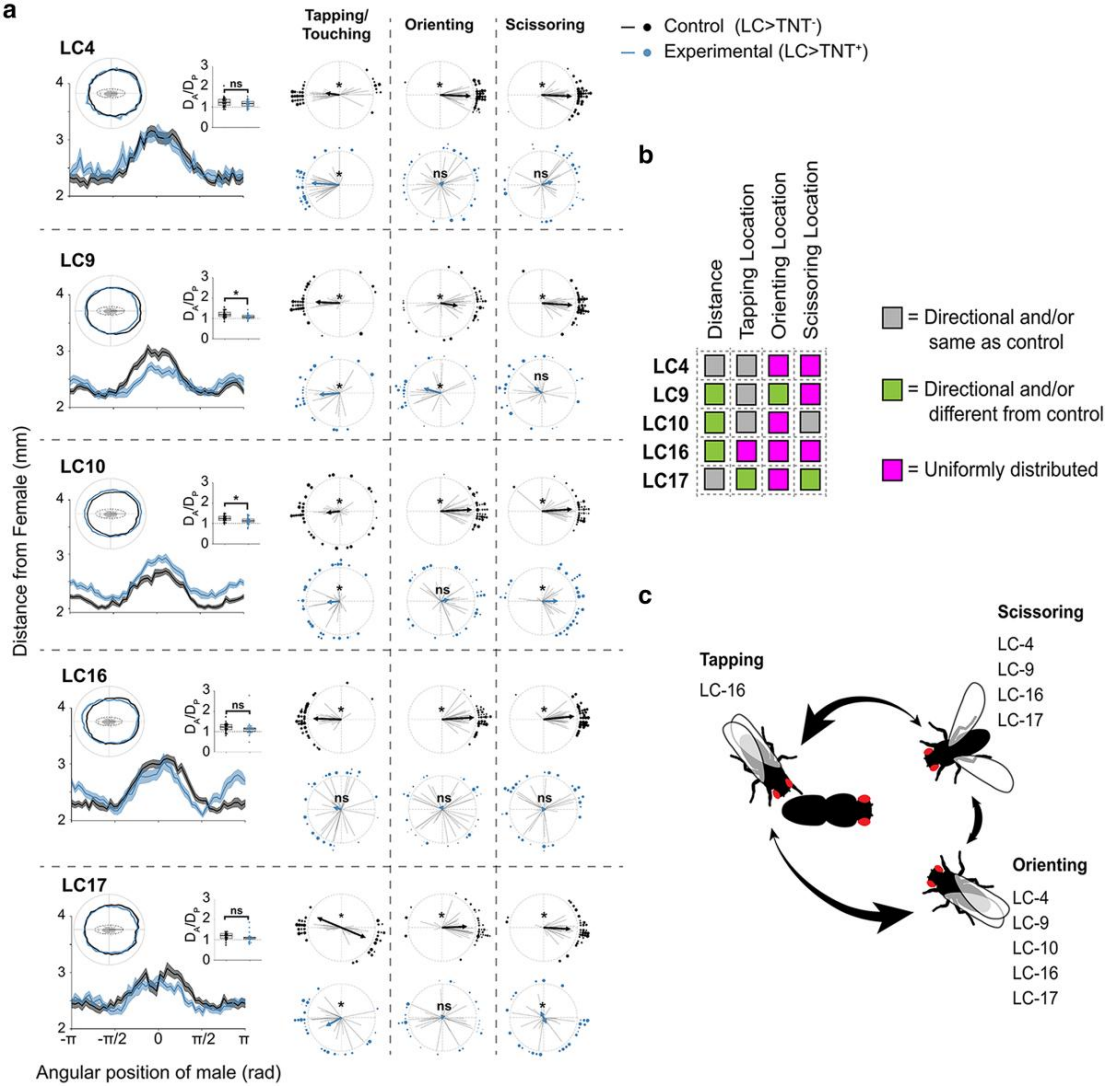
The spatiotemporal patterns of male courtship as a function of female body axis orientation depend on the activity of multiple independent visual projection neurons. a) Average courtship paths, ratios of the maximum male-to-female distance when a male is on either the anterior or posterior end of the female (*D_A_/D_P_*), and average angular positions of the male during Tapping/Touching, Orienting, and Scissoring are shown for each LC line expressing either an inactive (Control, *n* = 32 per line) or active (Experimental, *n* = 32 per line) version of the Tetanus toxin gene (TNT). Asterisks on *D_A_/D_P_* plots represent significant differences between Control and Experimental groups (*P <* 0.05, one-way ANOVA). Asterisks on circular plots highlight distributions that were significantly different from uniformity (*P <* 0.05, Rayleigh test or bimodal Rayleigh test). b) Gray and colored squares denote whether an LC line differed from controls in their spatial positioning during courtship following neural inactivation. c) Summary plot showing which LC lines were important for the proper spatial positioning of males during Tapping, Orienting, and Scissoring.

Animals were reared on a cornmeal-based food (Archon Scientific Inc) at 25 °C and 70% humidity under a 12h:12 h light:dark cycle. For behavioral assays, all flies were 4- to 6-day-old virgin males and females. Canton-S male and female flies were used for courtship experiments under white and red light (Figs. 1 and 2) and for decapitation and head-transplantation experiments (Fig. 3a–j). White-eyed females were derived from *w*^1118^ flies that had been back-crossed into Canton-S for at least 6 generations (*CS^w^*^–^), and these flies, along with their red-eyed Canton-S counterparts, were used as courtship targets in the red- vs white-eyed experiments (Fig. 3k–o). Canton-S females were used as courtship targets in the LC-inactivation experiments (Fig. 4), and males were derived from crosses between each LC-GAL4 line (LC4, LC9, LC10-1, LC16, LC17) and either an active (UAS-TNT^+^) or inactive (UAS-TNT^−^) mutant allele of the Tetanus Toxin gene (Sweeney et al. 1995; Wu et al. 2016).

### Courtship assay

All courtship trials were conducted at Zeitgeber time (ZT) 1 to 5, using 4- to 6 d-old virgin male and female flies. Both males and females were collected immediately following eclosion and moved into 25 mL plastic vials containing corn-meal-based food. Both males and females were kept in single-sex groups of 10 to 12 for 2 d, at which time individual males were moved into 5 mL glass vials containing a small amount of fly food and isolated for at least 2 additional days before testing. On test day, legs and wings were surgically removed from each female target, which was subsequently adhered to a rectangular piece of plastic weigh-boat (30mm × 30 mm) using UV-hardening glue (RapidFix). A circular courtship arena (23 mm diameter × 6 mm height) was placed over the fixed female, and males were aspirated into the chamber and allowed to freely court the female for 10 min. The orientation of the anterior-posterior body axis of each target female was random across trials.

### Tracking, classification, and machine-assisted analyses of male courtship behavior

Videos were recorded on a Raspberry Pi NoIR camera with a Navitar 8 to 48 mm lens for 10 min at 24 frames per second and were backlit using LEDs. To record under red-light conditions, LEDs were covered with long-pass, red filters (Neewer Inc.).

All videos were analyzed on a per-frame basis using custom software that tracks body and wing positions of courting flies and subsequently classifies whether the male was engaging in a particular behavior. Three simplified behavioral classifiers were created for identifying frames that contained males engaging in bouts of either (i) tapping, (ii) static orienting, or (iii) static wing scissoring. For each frame, several features were calculated from tracking data for use in an AdaBoost decision tree classifier (see Supplementary Table 1, in Supplementary File 1), based on both previous studies and an empirical classifier cross-validation protocol, which yielded greater accuracies (Freund and Schapire 1997; Branson et al. 2009; Kabra et al. 2012).

To eliminate the possible confounding effects of the various experimental manipulations on the ability of our algorithm to correctly identify each of the tracked behaviors, new classifiers for each of the courtship components measured here were generated *de novo* for each independent experimental dataset by first hand-scoring a subset of frames from at least 4 independent videos of control males and 4 videos experimental males. All classifiers had accuracies *>*95% (see Supplementary Table 1 for features used to generate behavioral classifiers, and Supplementary Table 2 for an example of classifier validation for control male courtship). To further improve classification accuracies, all videos were hand-scored for bouts of courtship, and any positive behavioral classifications falling outside these boundaries were discarded. For manual scoring, courtship was defined as any period in which the gaze of the male was directed toward the female, following the first observation of male wing extensions/scissoring.

Under these highly artificial behavioral conditions, classifications of each of the following 3 individual courtship elements in each frame were mutually exclusive. We achieved this by specifying an empirically determined phenotypic hierarchy whereby active “tapping” takes the highest precedence, followed by stationary “scissoring,” and then stationary “orienting.” Consequently, a single behavior was classified in each frame. This procedure was essential for generating robust differentiation between the highly correlated scissoring and orienting behaviors (see Fig. 1f).

#### Courtship path

The courtship path of each male was calculated by dividing the angular space surrounding the female into 50 bins and taking the mean centroid-to-centroid distance between the male and female during bouts of courtship. For some experiments, both control and experimental males attempted to copulate with the female for extended periods of time. While these males were not physically able to copulate since the female was fixed in place, these long durations of minimal movement had a significant effect on the courtship path, and for our purposes, represented bouts of copulation; they were therefore removed from all analyses.

#### Anterior-posterior distance ratio (*D_A_*/*D_P_*)

The *D_A_/D_P_* ratio was calculated as the ratio of the maximum courtship path when the male was on the front half of the female (−*π/*2 *< θ_male_ < π/*2) to when the male was on the rear half of the female (*θ_male_ <* −*π/*2 or *θ_male_ > π/*2).

#### Angular locations of courtship elements

The mean angular position of the male with respect to the female was calculated across all frames containing positive classifications for each behavior of interest. Rayleigh values (represented as arrow length in circular plots) were calculated for populations of males.

#### Bimodal Rayleigh test

Raw angular distributions were first compared to uniformity using the Rayleigh test. If no significant difference was found, we then tested for bimodality as follows. Angular distributions were transformed using the following equation, as in (Landler et al. 2018): *θ* = 2*t* mod 2*π*, where *t* are the original angles and *θ* are the transformed angles. This distribution was subsequently compared to the uniform distribution using a Rayleigh Test.

#### Courtship latency and index

The courtship latency was calculated as the time taken a male to start courting the female. Overall courtship index was calculated as the fraction of time a male spent courting a female during a 10-min trial (*t_courting_/t*_(*trial−duration*)_). We also calculated an index for each of the 3 courtship elements we observed in our paradigm, which represent the fraction of time a male spent performing a specific element with respect to the total courtship time (*t_element_/t_total_*).

## Results and discussion

### Vision contributes to stereotypic spatiotemporal patterns of male courtship

Female motion is an important visual cue used by male *Drosophila* to initiate and direct chase behaviors during bouts of courtship (Cook 1979; Agrawal et al. 2014; Kohatsu and Yamamoto 2015; Ribeiro et al. 2018). However, the visual cues utilized by males in the regulation of other spatial or temporal aspects of courtship elements is not as well understood. We hypothesized that males use anatomical features of courted females to direct spatiotemporal patterns of individual courtship elements. To separate the visual effects of the morphology of the female body from the well-established visual impact of her motion on the spatial patterns of male courtship, we first developed a simplified courtship paradigm that eliminates female motion-related visual cues. We then used a custom tracking and behavioral classification software to analyze the spatial localization of males during the release of specific elements of the courtship ritual (Fig. 1a).

We found that males take an asymmetric and stereotyped path around a stationary female during courtship whereby they positioned themselves ∼1.5 times further away from the head of the female than the tip of her abdomen (*P <* 0.001, 1-sample *T*-test; Fig. 1b and c). Because the reduced courtship assay we have used here is markedly different from the courtship assays, we (Ben-Shahar et al. 2010; Lu et al. 2012, 2014; Vernier et al. 2023) and others (Yamamoto et al. 2014; Benton 2015; Newell et al. 2016; McKellar et al. 2019) have used in the past, we first identified and characterized the specific courtship elements exhibited by wildtype Canton-S males. We found that in our assay, males primarily exhibited 3 distinct, easily classified courtship elements that accounted for ∼90% of the time males actively courted (Fig. 1d): (i) “Tapping,” which was defined as behaviors when the male is both courting the female and close enough to tap or lick her, (ii) “Orienting,” which was defined as periods of time when the angular heading of the male (gaze) is directed toward the female, the centroid of the male is static, and no wing extensions are observed, and (iii) “Scissoring,” which was defined as periods of time when the male was orienting and extending and vibrating at least one of his wings toward the female (Supplementary Table 1 and Supplementary Video 1). In our assay, scissoring was distinct from the single-wing extensions associated with the song produced by males when chasing an intact female (Sadaf et al. 2015; Koganezawa et al. 2016; Ding et al. 2019). However, studies of male-male social interactions in *D. melanogaster* and male-female courtship in related *Drosophila* species, indicate that wing scissoring is a natural behavioral element associated with inter-individual interactions in this animal clade (Cowling and Burnet 1981; Welbergen et al. 1987; Hoikkala and Crossley 2000; Fernandez et al. 2010; Higuchi et al. 2017). Although speculative, one possible explanation for why males exhibit increased scissoring in our reduced courtship assay is that when the female is fixed in space, the lack of motion by the courtship target triggers males to release auditory signals toward the anterior end of the female, which increases their perceived “quality,” and possibly motivate the female to allow the male to chase her. Such use of auditory signals has been reported for other species of *Drosophila* (Hoikkala et al. 1998).

We also analyzed the spatial patterns of each courtship element, which indicated that bouts of tapping occur when males are near the posterior half of the female, whereas bouts of orienting and scissoring occur when males are near the anterior half of the female (Fig. 1e). Accordingly, tapping positions are largely anti-correlated with scissoring and orienting positions, whereas orienting and scissoring positions were highly correlated with one another (Fig. 1f). These data indicate that males use information about the anterior-posterior body axis of females to make courtship decisions.

We next sought to determine whether males are using vision to determine the body axis position of courted females (Fig. 2). We found that in contrast to courting under white light (intact vision), males courting under red light (limited vision) position themselves closer to the anterior end of females (*P <* 0.001, 1-sample *T*-test; Fig. 2a–c), tap females on both the posterior and anterior ends (*P <* 0.001, Bimodal Rayleigh test; Fig. 2d), and orient toward the posterior end of females (*P <* 0.05, Rayleigh test; Fig. 2d). Similar to tapping, scissoring followed a bimodal distribution under red light conditions (*P <* 0.05 Bimodal Rayleigh Test; Fig. 2d). Furthermore, we found that although red light conditions reduce the overall courtship index of males, the relative time spent tapping is increased (*P <* 0.05, Kruskal test), scissoring is decreased (*P <* 0.05, Kruskal test), and orienting remained constant (Fig. 2e–g). These data suggest that although vision is not required for courtship in general, nor for the specific release of any of its individual elements, it is important for directing spatiotemporal aspects of courtship displays. Furthermore, these data show that males can compensate for the loss of visual sensory information by increasing chemo- and/or tactile sensory inputs associated with tapping.

### Males use the eyes of females as a visual marker for directing the spatial distributions of specific courtship elements

Having established that vision contributes to the regulation of the spatiotemporal patterns of male courtship in our assay, we next asked which specific morphological features of the female anterior-posterior body axis might serve to visually guide males during courtship. Specifically, because the red-pigmented eyes of flies are highly contrasted with the lighter-colored cuticle, we hypothesized that they could serve as a robust visual cue males use to mark the anterior end of the body of courted females (Supplementary Fig. 1a and b).

To test this hypothesis, we first asked whether the head of females is necessary for regulating any spatial aspects of the male courtship ritual (Fig. 3a–e and Supplementary Fig. 1c–e). We found that when courting headless females, male courtship paths are symmetric and equidistant from either end of the antero-posterior body axis of females (Fig. 3a–d), and the mean angular positions of tapping, orienting, and scissoring behaviors are either bimodally or uniformly distributed around the female (*P <* 0.05 for tapping, Bimodal Rayleigh test; *P >* 0.05 for orienting and scissoring, Rayleigh test; Fig. 3e). While the overall courtship latencies and indices are unaffected by headless females (Supplementary Fig. 1c and d), we did observe an overall decrease in scissoring frequency, suggesting that the female head is particularly important for visually triggering its release (*P <* 0.05, Kruskal test; Supplementary Fig. 1e).

Next, to determine whether visual cues associated with the female head are sufficient for regulating the spatiotemporal patterns of individual courtship elements, we examined the behavior of males courting females whose head has been transplanted from the anterior to posterior end. We found that although head position has no effect on the overall levels of courtship, or on the frequencies of expressing individual courtship elements (Supplementary Fig. 1f–h), males courting females with a posterior head position (“Head-Posterior”) exhibit an asymmetric courtship path that is biased toward greater distances from the posterior rather than the anterior end of the female (Fig. 3f–i). Furthermore, we found that when courting “Head-Posterior” females, males tend to tap the anterior end of the female body axis. In contrast, the spatial distributions of orienting and scissoring toward “Head-Posterior” females are randomly distributed (tapping: *P <* 0.05, Rayleigh test; orienting and scissoring: *P >* 0.05, Rayleigh test; Fig. 3j). Together, these results indicate that males use the female head as a visual marker for determining some aspects the anterior-posterior body axis orientation of courted females. However, visual recognition of the female head is not sufficient for driving the precise spatial patterns of orienting or scissoring behaviors.

Because the eyes are one of the primary features of the fly head, we next hypothesized that males specifically use the eyes of females as a visual landmark to determine the antero-posterior body axis of their courtship targets. To test this hypothesis, we generated 2 congenic lines of wildtype flies that differed in a single mutation in the *white* gene, resulting in red- and white-eyed females with inverted visual contrasts made between the eyes and surrounding cuticle (Supplementary Fig. 1a and b). Previous studies have suggested that changes in luminance are sufficient for stimulating majority of photoreceptors in the fly, and likely play a role in the visual perception of motion and edges (Behnia et al. 2014; Serbe et al. 2016), but are less important for detecting looming signals (de Vries and Clandinin 2012) or color perception (Yamaguchi et al. 2008). However, whether the fly visual system is specifically tuned for detecting the specific luminance values and contrast represented by red eyes is not well understood. We found that although both female genotypes elicited asymmetric courtship paths, the distances of males from the anterior end of white-eyed females were significantly reduced when compared to males courting red-eyed females (*P <* 0.05, Student's *T*-test; Fig. 3k–n). Further, we observed that while males courting white-eyed females tapped mostly at the posterior end, the mean angular locations of both orienting and scissoring bouts were bimodally distributed around the antero-posterior axis of the female (*P <* 0.001 for tapping, Rayleigh Test; *P <* 0.001 for orienting and scissoring, Bimodal Rayleigh Test; Fig. 3o). In addition, while the relative durations of tapping and orienting were not affected by female eye color, we found that males courting white-eyed females spent significantly less time scissoring (*P <* 0.01, Kruskal test; Supplementary Fig. 1i–k). These data suggest that males are possibly using the eyes of females as one of the visual landmarks for recognizing the anterior-posterior body axis of their courtship targets. Nonetheless, at least some spatial aspects of the male courtship ritual were retained when males courted white-eyed females, which suggest that additional features of the female body contribute visual information important for determining body axis orientation. For example, males could also use the cuticular banding pattern on the abdomen of a target female as a landmark for her posterior end, especially as pigmentation patterns have been shown to be under strong sexual selection (Protas and Patel 2008; Wittkopp and Beldade 2009).

### Visually driven spatiotemporal aspects of the male courtship ritual depend on the activity of multiple independent populations of visual projection neurons

Several recent studies have indicated that ectopic activation of specific classes of Lobula Columnar (LC) visual projection neurons is sufficient to trigger various visually-guided behaviors (Wu et al. 2016; Sen et al. 2017), including motion detection in courtship (Ribeiro et al. 2018). These data suggest that innate responses to some visual stimuli are mediated via modular and stereotypic circuit elements. Consequently, because our behavioral data suggest that males are likely using visual landmarks to define the anterior–posterior body axis of courted females, we next wanted to know whether this capacity depends on a relatively simple integration of one or very few visual elements, or a more complex integration of many different features, via multiple independent LC-dependent pathways.

Based on previously published data, we chose to focus our investigation on 4 classes of LC neurons that could be involved in regulating various courtship behaviors, including: leg reaching (LC10), forward walking (LC17), backward walking (LC9, LC10, LC16, LC17), and turning (LC16, LC17) (Sen et al. 2017). We additionally chose to examine LC4 neurons because they have been shown to function in detecting looming stimuli (Wu et al. 2016; von Reyn et al. 2017), which is a visual feature likely to be encountered by males as they approach female courtship targets. We found that synaptic silencing of these populations of LC neurons by using targeted transgenic expression of tetanus toxin (TNT) (Sweeney et al. 1995) affected at least one spatiotemporal aspect of the male courtship ritual when compared with LC lines that express the inactive TNT allele. Specifically, we found that while tapping patterns remained largely intact, the spatial patterns of both orienting and scissoring were mostly uniformly or bimodally distributed around the female following LC inactivation. However, for reasons we do not understand, control LC17 animals showed abnormal bimodal tapping pattern at baseline (Fig. 4a). Nevertheless, these data suggest that the regulation of orienting and scissoring behaviors is more reliant on precise visual signaling through LC neurons than tapping. In addition to spatial deficits, we found that inhibiting individual LC neuron classes had varying effects on temporal aspects of the courtship ritual, including regulating the latency to court, the fraction of time spent courting, and the relative fractions of time spent exhibiting individual courtship elements (Supplementary Fig. 2). Although the primary goal of this analysis was not to map the specific circuits that drive male courtship decisions, our data indicate that the correct spatiotemporal release of at least some of the courtship elements we observed in our reduced courtship paradigm depends on complex activity patterns of multiple classes of LC neurons, each of which likely integrates a limited set of visual features present on the courted female.

We also examined the impact of silencing individual LC neurons on the average velocity of males at different angular positions around female courtship targets (Supplementary Fig. 3a). In our hands, the parameter that showed the most robust changes in our reduced paradigm was sideway velocity of the male (Supplementary Fig. 3a). We found that control males exhibited stereotypical movement patterns, where they accelerated to high velocities when on either side of the medial-lateral axis of the female (*V_ML_*, areas shown in green in Supplementary Fig. 3b), and then slowed down significantly when either on the anterior (*V_A_*) or posterior (*V_P_*) ends of the female. In contrast, inactivation of most LC neurons resulted in slower overall velocities and a failure to slow down when reaching either the anterior or posterior ends of the courted female (Supplementary Fig. 3d). We observed a similar effect on velocity for males courting headless females (Supplementary Fig. 3e–g), which suggests that at least some LC neurons regulate changes in velocity via visual recognition of the female head.

Together, these data suggest that males use a relatively simple visual cue to define the anteroposterior body axis of their courtship targets. Yet, the precise spatial and temporal release of specific courtship elements in response to this cue seems to depend on the complex activities of multiple independent populations of LC neurons. While we currently do not know how these circuits interact to generate specific behavioral outputs in the context of courtship, it is likely that each population of visual projection neurons responds to a limited set of visual features, and the collective activity of these neurons is then integrated with other contextual cues to generate an optimal courtship pattern. For instance, when a male approaches a stationary female during courtship, she would appear to be looming, a visual feature which has been previously shown to be detected by LC4 and LC16 neurons in the context of eliciting escape behaviors (Wu et al. 2016; Sen et al. 2017; von Reyn et al. 2017; Ache et al. 2019). Our data indicate that under different behavioral contexts, some LC neurons can elicit different motor outputs in response to homologous visual stimuli, which suggests that they might interact with multiple independent, downstream behavior-specific circuits.

Here we have shown that male *Drosophila* use anatomical features of the female body to regulate spatiotemporal aspects of their courtship ritual. Specifically, we show that independent of female motion, males use the eyes of females as an indicator of her anterior end. Nonetheless, our data also indicate that eyes are not the sole visual cue, nor vision is the only sensory modality used by males, to establish the anterior-posterior axis if courted females. We also do not understand yet what might be the relationship between the specific visual cues used in our highly reduced assay relative to courtship behavior exhibited by males when chasing an intact female. Regardless, our data clearly show that males are capable of visually determining the anterior-posterior axis of females and use this information to regulate the spatiotemporal patterns of their courtship. Furthermore, we demonstrate that although male courtship is considered an innate, “hardwired” behavior, it includes a putative “optimization function”, which allows males to maximize their reproductive success when facing sub-optimal environmental conditions that constrain the available sensory information, as has been shown for *Drosophila* courtship conditioning and mating success in other insect species (Joiner and Griffith 2000; Certel et al. 2010; Fowler-Finn and Rodríguez 2012). Therefore, it is not surprising that when males have limited access to visual inputs, they decrease the amount of time spent scissoring, and increase the amount of time spent tapping, possibly suggesting that males are compensating for the lack of visual information by increasing pheromonal and tactile sensory inputs.

Finally, we demonstrated that some visual projection neurons function as modular integrators, which can engage different downstream output modules based on the current behavioral context of an individual. For example, specific looming-detection neurons might elicit an escape response in some situations while also helping males determine the body axis of courted females under different circumstances. Although we do not understand yet how similar sensory cues can elicit contextual behavior, we anticipate that future studies will reveal that this form of context-specific circuit modularity is a common feature of the visual system, and sensory systems in general.

### Fly stocks

All fly stocks and other reagents used for these studies can be found in the supplementary Reagents Table file.

## Supplementary Material

jkag037_Supplementary_Data

## Data Availability

All software and scripts used for tracking, classification, and data analysis are publicly available at 10.5281/zenodo.18274754. Supplemental material available at G3 online.

## References

[jkag037-B1] Ache JM et al 2019. Neural basis for looming size and velocity encoding in the *Drosophila* giant fiber escape pathway. Curr Biol. 29:1073–1081.e4. 10.1016/j.cub.2019.01.079. 30827912

[jkag037-B2] Agrawal S, Safarik S, Dickinson M. 2014. The relative roles of vision and chemosensation in mate recognition of *Drosophila melanogaster*. J Exp Biol. 217:2796–2805. 10.1242/jeb.105817. 24902744

[jkag037-B3] Baker CA et al 2022. Neural network organization for courtship-song feature detection in *Drosophila*. Curr Biol. 32:3317–3333.e7. 10.1016/j.cub.2022.06.019. 35793679 PMC9378594

[jkag037-B4] Behnia R, Clark DA, Carter AG, Clandinin TR, Desplan C. 2014. Processing properties of ON and OFF pathways for *Drosophila* motion detection. Nature. 512:427–430. 10.1038/nature13427. 25043016 PMC4243710

[jkag037-B5] Ben-Shahar Y et al 2010. The Drosophila gene CheB42a is a novel modifier of Deg/ENaC channel function. PLoS One. 5:e9395. 10.1371/journal.pone.0009395. 20195381 PMC2827562

[jkag037-B6] Benton R . 2015. Neural circuits: male mating motifs. Neuron. 87:912–914. 10.1016/j.neuron.2015.08.017. 26335638

[jkag037-B7] Branson K, Robie AA, Bender J, Perona P, Dickinson MH. 2009. High-throughput ethomics in large groups of *Drosophila*. Nat Methods. 6:451–457. 10.1038/nmeth.1328. 19412169 PMC2734963

[jkag037-B8] Certel SJ et al 2010. Octopamine neuromodulatory effects on a social behavior decision-making network in *Drosophila* males. PLoS One. 5:e13248. 10.1371/journal.pone.0013248. 20967276 PMC2953509

[jkag037-B9] Clowney EJ, Iguchi S, Bussell JJ, Scheer E, Ruta V. 2015. Multimodal chemosensory circuits controlling male courtship in *Drosophila*. Neuron. 87:1036–1049. 10.1016/j.neuron.2015.07.025. 26279475 PMC4560615

[jkag037-B10] Cook R . 1979. The courtship tracking of *Drosophila melanogaster*. Biol Cybern. 34:91–106. 10.1007/BF00365473.

[jkag037-B11] Cowling DE, Burnet B. 1981. Courtship songs and genetic control of their acoustic characteristics in sibling species of the *Drosophila melanogaster* subgroup. Anim Behav. 29:924–935. 10.1016/S0003-3472(81)80030-9.

[jkag037-B12] de Vries SEJ, Clandinin TR. 2012. Loom-sensitive neurons link computation to action in the *Drosophila* visual system. Curr Biol. 22:353–362. 10.1016/j.cub.2012.01.007. 22305754 PMC3298569

[jkag037-B13] Ding Y, Lillvis JL. 2025. Neural evolution of complex motor behaviors: insights from *Drosophila* courtship song. Curr Opin Neurobiol. 95:103131. 10.1016/j.conb.2025.103131.41172557

[jkag037-B14] Ding Y et al 2019. Neural evolution of context-dependent fly song. Curr Biol. 29:1089–1099.e7. 10.1016/j.cub.2019.02.019. 30880014

[jkag037-B15] Ejima A, Griffith LC. 2008. Courtship initiation is stimulated by acoustic signals in *Drosophila melanogaster*. PLoS One. 3:e3246. 10.1371/journal.pone.0003246. 18802468 PMC2531232

[jkag037-B16] Fedotov SA et al 2026. The structure of courtship behavior in *Drosophila* males: boundaries of plasticity. Behav Processes. 234:105312. 10.1016/j.beproc.2025.105312. 41325927

[jkag037-B17] Fernandez MP et al 2010. Pheromonal and behavioral cues trigger male-to-female aggression in *Drosophila*. PLoS Biol. 8:e1000541. 10.1371/journal.pbio.1000541. 21124886 PMC2990703

[jkag037-B18] Fowler-Finn KD, Rodríguez RL. 2012. Experience-mediated plasticity in mate preferences: mating assurance in a variable environment. Evolution. 66:459–468. 10.1111/j.1558-5646.2011.01446.x. 22276541

[jkag037-B19] Freund Y, Schapire RE. 1997. A decision-theoretic generalization of on-line learning and an application to boosting. J Comput Syst Sci. 55:119–139. 10.1006/jcss.1997.1504.

[jkag037-B20] Higuchi T, Kohatsu S, Yamamoto D. 2017. Quantitative analysis of visually induced courtship elements in *Drosophila subobscura*. J Neurogenet. 31:49–57. 10.1080/01677063.2017.1290613. 28552034

[jkag037-B21] Hindmarsh Sten T, Li R, Otopalik A, Ruta V. 2021. Sexual arousal gates visual processing during *Drosophila* courtship. Nature. 595:549–553. 10.1038/s41586-021-03714-w. 34234348 PMC8973426

[jkag037-B22] Hoikkala A, Aspi J, Suvanto L. 1998. Male courtship song frequency as an indicator of male genetic quality in an insect species, *Drosophila* montana. Proc R Soc Lond B Biol Sci. 265:503–508. 10.1098/rspb.1998.0323. PMC1688912 9569668

[jkag037-B23] Hoikkala A, Crossley S. 2000. Copulatory courtship in *Drosophila*: behavior and songs of *D. birchii* and *D. serrata*. J Insect Behav. 13:71–86. 10.1023/A:1007715609756.

[jkag037-B24] Joiner MA, Griffith LC. 2000. Visual input regulates circuit configuration in courtship conditioning of *Drosophila melanogaster*. Learn Mem. 7:32–42. 10.1101/lm.7.1.32. 10706600 PMC311320

[jkag037-B25] Kabra M, Robie AA, Rivera-Alba M, Branson S, Branson K. 2012. JAABA: interactive machine learning for automatic annotation of animal behavior. Nat Methods. 10:64–67. 10.1038/nmeth.2281. 23202433

[jkag037-B26] Koganezawa M, Kimura K-I, Yamamoto D. 2016. The neural circuitry that functions as a switch for courtship versus aggression in *Drosophila* males. Curr Biol. 26:1395–1403. 10.1016/j.cub.2016.04.017 27185554

[jkag037-B27] Kohatsu S, Yamamoto D. 2015. Visually induced initiation of *Drosophila* innate courtship-like following pursuit is mediated by central excitatory state. Nat Commun. 6:6457. 10.1038/ncomms7457. 25743851

[jkag037-B28] Krstic D, Boll W, Noll M. 2009. Sensory integration regulating male courtship behavior in *Drosophila*. PLoS One. 4:e4457. 10.1371/journal.pone.0004457. 19214231 PMC2636894

[jkag037-B29] Landler L, Ruxton GD, Malkemper EP. 2018. Circular data in biology: advice for effectively implementing statistical procedures. Behav Ecol Sociobiol. 72:128. 10.1007/s00265-018-2538-y. 30100666 PMC6060829

[jkag037-B30] Leitner N, Ben-Shahar Y. 2020. The neurogenetics of sexually dimorphic behaviors from a postdevelopmental perspective. Genes Brain Behav. 19:e12623. 10.1111/gbb.12623. 31674725

[jkag037-B31] Lu B, LaMora A, Sun Y, Welsh MJ, Ben-Shahar Y. 2012. ppk23-dependent chemosensory functions contribute to courtship behavior in *Drosophila melanogaster*. PLoS Genet. 8:e1002587. 10.1371/journal.pgen.1002587. 22438833 PMC3305452

[jkag037-B32] Lu B, Zelle KM, Seltzer R, Hefetz A, Ben-Shahar Y. 2014. Feminization of pheromone-sensing neurons affects mating decisions in *Drosophila* males. Biol Open. 3:152–160. 10.1242/bio.20147369. 24463366 PMC3925318

[jkag037-B33] Markow TA . 1987. Behavioral and sensory basis of courtship success in *Drosophila melanogaster*. Proc Natl Acad Sci U S A. 84:6200–6204. 10.1073/pnas.84.17.6200. 3114743 PMC299038

[jkag037-B34] McKellar CE et al 2019. Threshold-based ordering of sequential actions during *Drosophila* courtship. Curr Biol. 29:426–434.e6. 10.1016/j.cub.2018.12.019. 30661796

[jkag037-B35] Moon SJ, Lee Y, Jiao Y, Montell C. 2009. A *Drosophila* gustatory receptor essential for aversive taste and inhibiting male-to-male courtship. Curr Biol. 19:1623–1627. 10.1016/j.cub.2009.07.061. 19765987 PMC2762023

[jkag037-B36] Newell NR, New FN, Dalton JE, McIntyre LM, Arbeitman MN. 2016. Neurons that underlie *Drosophila melanogaster* reproductive behaviors: detection of a large male-bias in gene expression in fruitless-expressing neurons. G3 (Bethesda). 6:2455–2465. 10.1534/g3.115.019265. 27247289 PMC4978899

[jkag037-B37] Ning J et al 2022. Behavioral signatures of structured feature detection during courtship in *Drosophila*. Curr Biol. 32:1211–1231. e7. 10.1016/j.cub.2022.01.024. 35139360

[jkag037-B38] Pavlou HJ, Goodwin SF. 2013. Courtship behavior in *Drosophila melanogaster*: towards a ‘courtship connectome’. Curr Opin Neurobiol. 23:76–83. 10.1016/j.conb.2012.09.002. 23021897 PMC3563961

[jkag037-B39] Protas ME, Patel NH. 2008. Evolution of coloration patterns. Annu Rev Cell Dev Biol. 24:425–446. 10.1146/annurev.cellbio.24.110707.175302. 18593352

[jkag037-B40] Ribeiro IMA et al 2018. Visual projection neurons mediating directed courtship in *Drosophila*. Cell. 174:607–621.e18. 10.1016/j.cell.2018.06.020. 30033367

[jkag037-B41] Rings A, Goodwin SF. 2019. To court or not to court–a multimodal sensory decision in *Drosophila* males. Curr Opin Insect Sci. 35:48–53. 10.1016/j.cois.2019.06.009. 31336357

[jkag037-B42] Sadaf S, Reddy OV, Sane SP, Hasan G. 2015. Neural control of wing coordination in flies. Curr Biol. 25:80–86. 10.1016/j.cub.2014.10.069. 25496964

[jkag037-B43] Schretter CE et al 2025. Social state alters vision using three circuit mechanisms in *Drosophila*. Nature. 637:646–653. 10.1038/s41586-024-08255-6. 39567699 PMC11735400

[jkag037-B44] Seeholzer LF, Seppo M, Stern DL, Ruta V. 2018. Evolution of a central neural circuit underlies *Drosophila* mate preferences. Nature. 559:564–569. 10.1038/s41586-018-0322-9. 29995860 PMC6276375

[jkag037-B45] Sen R et al 2017. Moonwalker descending neurons mediate visually evoked retreat in *Drosophila*. Curr Biol. 27:766–771. 10.1016/j.cub.2017.02.008. 28238656

[jkag037-B46] Serbe E, Meier M, Leonhardt A, Borst A. 2016. Comprehensive characterization of the major presynaptic elements to the *Drosophila* OFF motion detector. Neuron. 89:829–841. 10.1016/j.neuron.2016.01.006. 26853306

[jkag037-B47] Shiozaki HM et al 2024. Activity of nested neural circuits drives different courtship songs in *Drosophila*. Nat Neurosci. 27:1954–1965. 10.1038/s41593-024-01738-9. 39198658 PMC11452343

[jkag037-B48] Sweeney ST, Broadie K, Keane J, Niemann H, O'Kane CJ. 1995. Targeted expression of tetanus toxin light chain in *Drosophila* specifically eliminates synaptic transmission and causes behavioral defects. Neuron. 14:341–351. 10.1016/0896-6273(95)90290-2. 7857643

[jkag037-B49] Thistle R, Cameron P, Ghorayshi A, Dennison L, Scott K. 2012. Contact chemoreceptors mediate male-male repulsion and male-female attraction during *Drosophila* courtship. Cell. 149:1140–1151. 10.1016/j.cell.2012.03.045. 22632976 PMC3365544

[jkag037-B50] Toda H, Zhao X, Dickson BJ. 2012. The *Drosophila* female aphrodisiac pheromone activates ppk23+ sensory neurons to elicit male courtship behavior. Cell Rep 1:599–607. 10.1016/j.celrep.2012.05.007. 22813735

[jkag037-B51] Vernier CL et al 2023. A pleiotropic chemoreceptor facilitates the production and perception of mating pheromones. iScience. 26:105882. 10.1016/j.isci.2022.105882. 36691619 PMC9860498

[jkag037-B52] Vijayan V, Thistle R, Liu T, Starostina E, Pikielny CW. 2014. *Drosophila* pheromone-sensing neurons expressing the ppk25 ion channel subunit stimulate male courtship and female receptivity. PLoS Genet. 10:e1004238. 10.1371/journal.pgen.1004238. 24675786 PMC3967927

[jkag037-B53] Villella A, Hall JC. 2008. Neurogenetics of courtship and mating in *Drosophila*. Adv Genet. 62:67–184. 10.1016/S0065-2660(08)00603-2. 19010254

[jkag037-B54] von Reyn CR et al 2017. Feature integration drives probabilistic behavior in the *Drosophila* escape response. Neuron. 94:1190–1204.e1196. 10.1016/j.neuron.2017.05.036. 28641115

[jkag037-B55] Welbergen P, Scharloo W, Dijken FRV. 1987. Collation of the courtship behaviour of the sympatric Species *Drosophila melanogaster* and *Drosophila* simulans. Behaviour. 101:253–274. 10.1163/156853987X00017

[jkag037-B56] Willmund R, Ewing A. 1982. Visual signals in the courtship of *Drosophila melanogaster*. Anim Behav. 30:209–215. 10.1016/S0003-3472(82)80256-X.

[jkag037-B57] Wittkopp PJ, Beldade P. 2009. Development and evolution of insect pigmentation: genetic mechanisms and the potential consequences of pleiotropy. Semin Cell Dev Biol. 20:65–71. 10.1016/j.semcdb.2008.10.002. 18977308

[jkag037-B58] Wu M et al 2016. Visual projection neurons in the *Drosophila* lobula link feature detection to distinct behavioral programs. Elife. 5:e21022. 10.7554/eLife.21022. 28029094 PMC5293491

[jkag037-B59] Yamaguchi S, Wolf R, Desplan C, Heisenberg M. 2008. Motion vision is independent of color in *Drosophila*. Proc Natl Acad Sci U S A. 105:4910–4915. 10.1073/pnas.0711484105. 18353989 PMC2290790

[jkag037-B60] Yamamoto D, Sato K, Koganezawa M. 2014. Neuroethology of male courtship in *Drosophila*: from the gene to behavior. J Comp Physiol A Neuroethol Sens Neural Behav Physiol. 200:251–264. 10.1007/s00359-014-0891-5. 24567257

[jkag037-B62] Zhang Y, Ng R, Neville MC, Goodwin SF, Su CY. 2020. Distinct roles and synergistic function of Fru(M) isoforms in *Drosophila* olfactory receptor neurons. Cell Rep. 33:108516. 10.1016/j.celrep.2020.108516. 33326795 PMC7845487

