# Supplementary material for: Visual recognition of the anteroposterior female body axis drives spatial elements of male courtship in Drosophila
Source: G3 (Bethesda). 2026 Feb 16;16(4):jkag037. doi: 10.1093/g3journal/jkag037 (PMC13042310; doi:10.1093/g3journal/jkag037)
Supplement: jkag037_Supplementary_Data [file jkag037_supplementary_data.zip › Supplemental_Figures_and_Tables_G3-2025-406440.pdf]

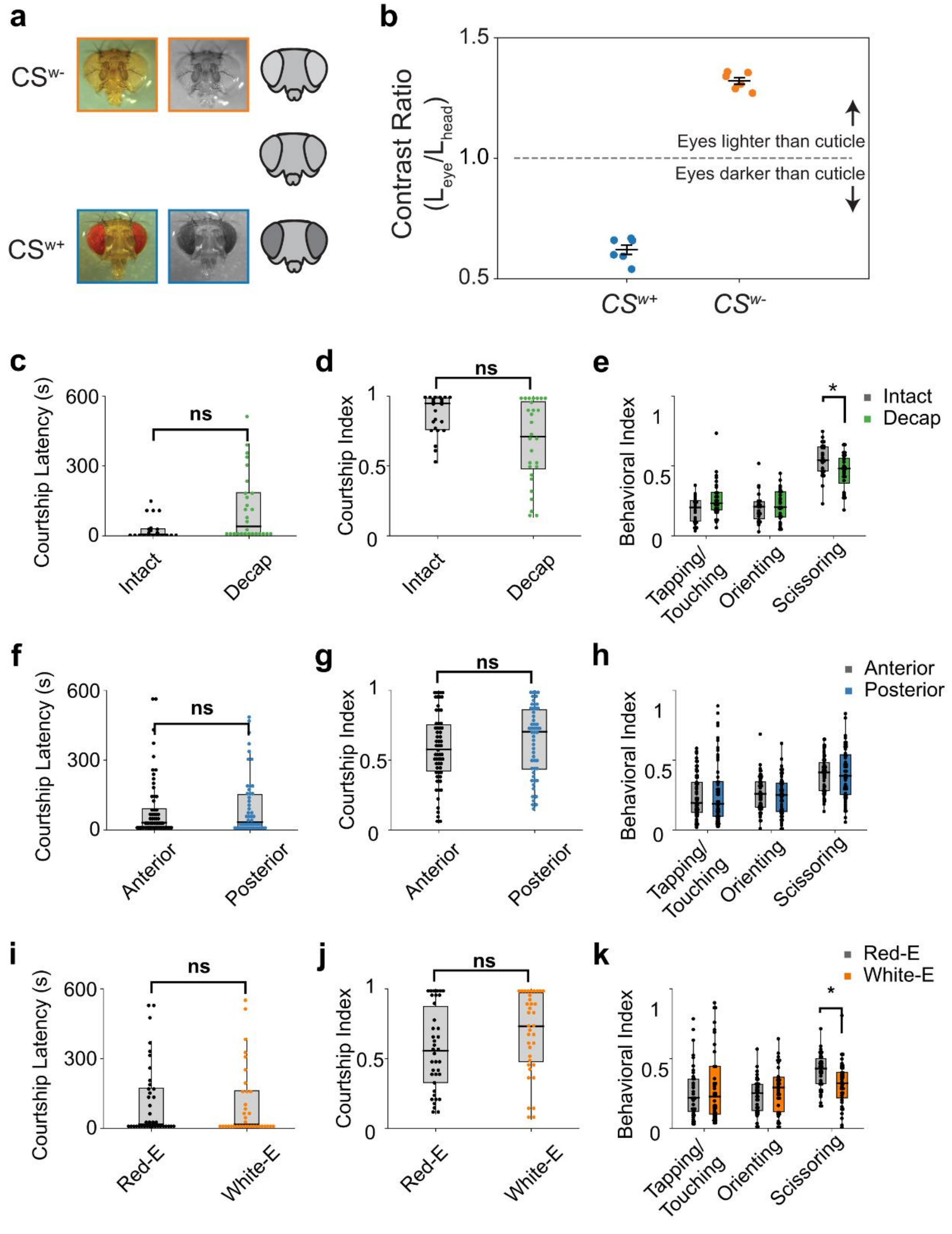

Figure S1. Female head, head position, and eye color regulate various temporal aspects of male courtship, Related to Figure 3.

a) Images of the heads of Canton-S (CS^{w+}, Red-E) and congenic females with a single mutation in the *white* gene (CS^{w-}, White-E) are shown next to a schematic of flies with varying eye contrasts.

b) Contrast ratios for CS^{w+} and CS^{w-} flies (n=6/group). Ratios were calculated by converting images to luminances and comparing pixels comprising the eyes (L_{eye}) to pixels comprising the cuticle surrounding the eyes (L_{head}).

c) Courtship latency for males courting intact (Intact) and decapitated (Decap) females.

d) Courtship index for males courting intact (Intact) and decapitated (Decap) females.

e) Behavioral indices for Tapping, Orienting, and Scissoring for males courting Intact and Decap females. Males courting Decap females scissor less than males courting Intact females ($p < 0.05$, Kruskal Test).

f) Courtship latency for males courting intact females (Anterior) and females that had their heads transplanted to their posterior end (Posterior).

g) Courtship index for males courting intact females (Anterior) and females that had their heads transplanted to their posterior end (Posterior).

h) Behavioral indices for Tapping, Orienting, and Scissoring for males courting Anterior and Posterior females.

i) Courtship latency for males courting red eyed (Red-E) and white eyed (White-E) females.

j) Courtship index for males courting red eyed (Red-E) and white eyed (White-E) females.

k) Behavioral indices for Tapping, Orienting, and Scissoring for males courting Red-E and White-E females. Males courting White-E females scissor less than males courting Red-E females ($p < 0.05$, Kruskal Test).

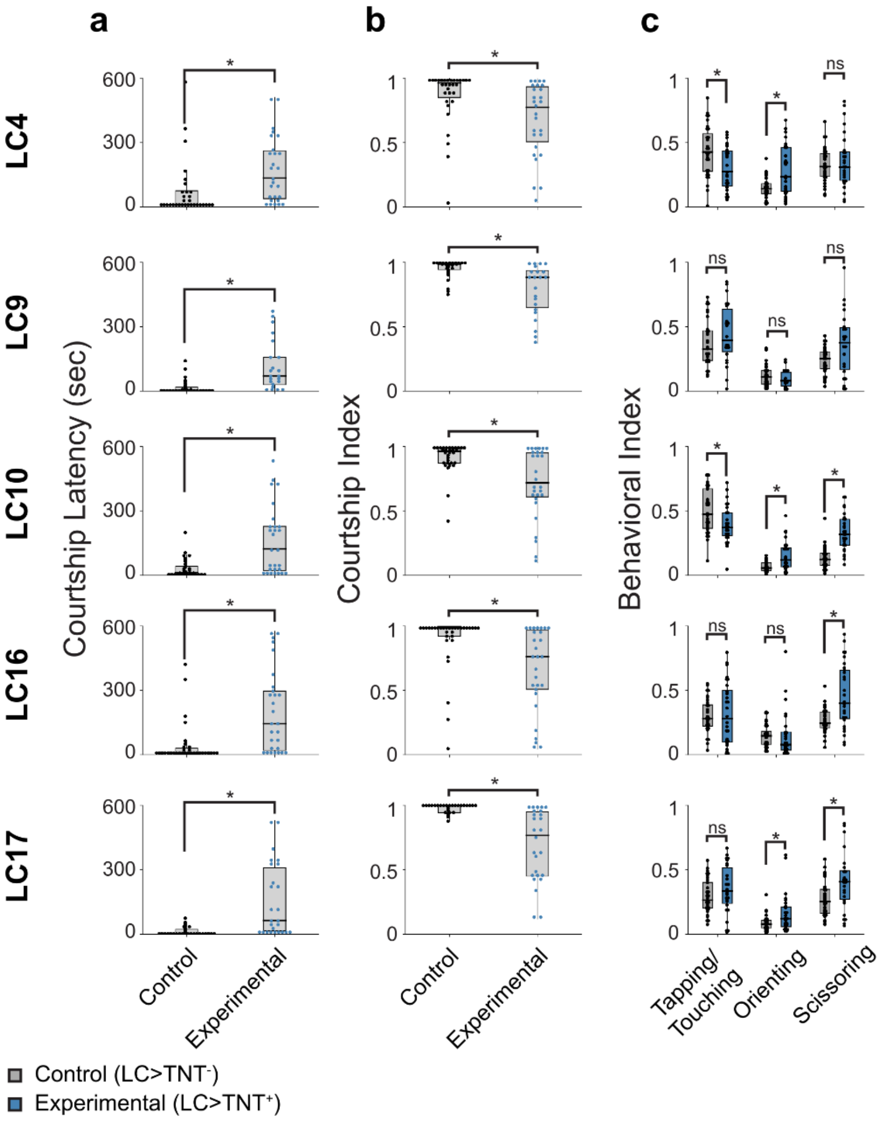

Figure S2. LC neurons are required for temporal aspects of the male courtship ritual, Related to Figure 4.

a) Inactivation of all LC neuron populations led to significant increases in courtship latencies when compared to controls (n=32/group, p < 0.05, Kruskal Test).

b) Inactivation of all LC neuron populations led to significant decreases in courtship indices when compared to controls (n=32/group, p < 0.05, Kruskal Test).

c) Behavioral indices for Tapping/Touching, Orienting, and Scissoring are shown for each LC neuronal population inactivation. Asterisks represent significant differences between groups (p<0.05, Kruskal Test).

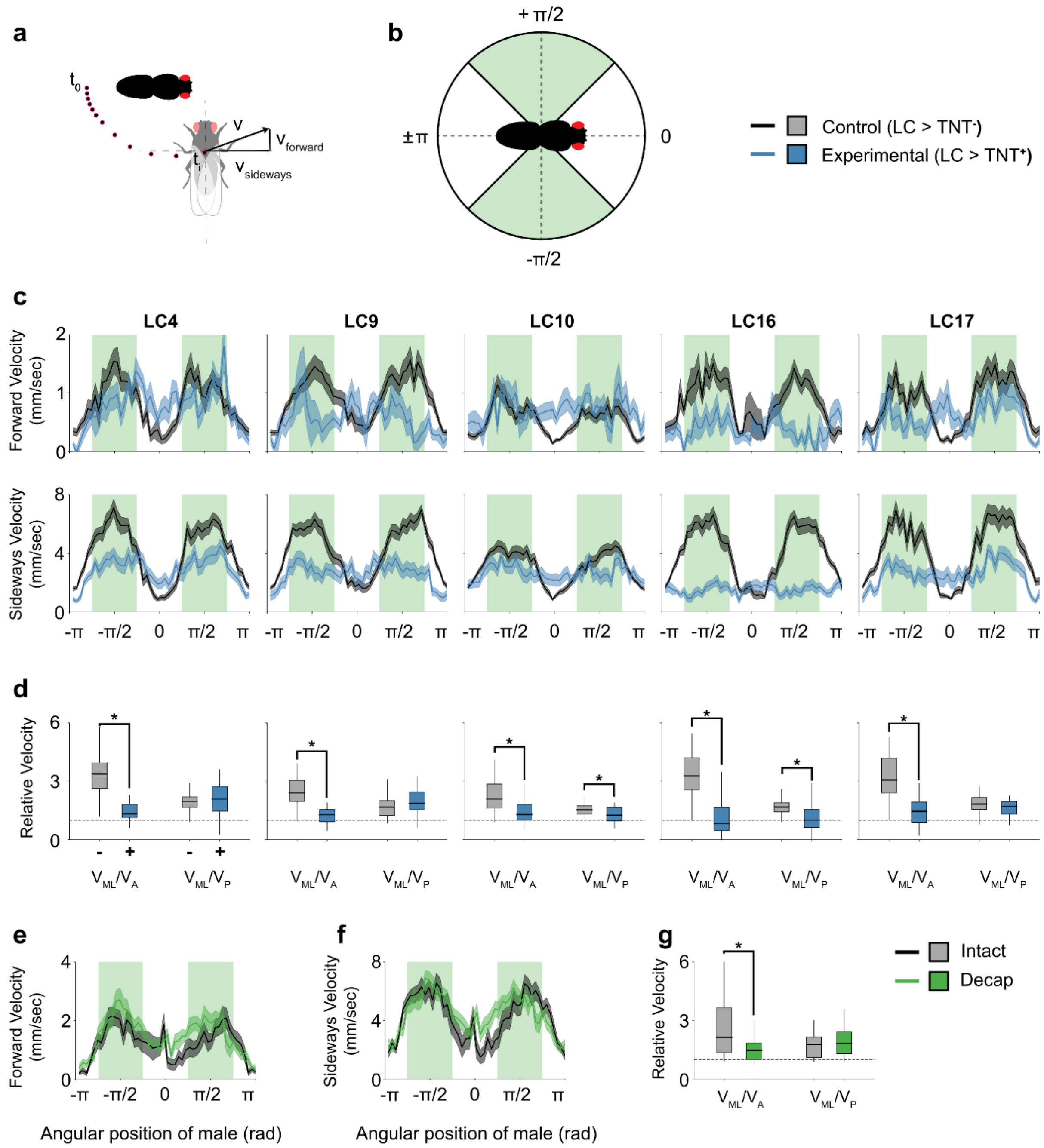

Figure S3. Visual projection neurons mediate movements during courtship, Related to Figure 3 and Figure 4.

a) Schematic showing the breakdown of the male's velocity vector (v) at a specific time point (t_i) into forward ($v_{forward}$) and sideways ($v_{sideways}$) components.

b) Schematic highlighting the four spatial quadrants surrounding the female.

c) Line plots showing the average velocity (±SEM) of each population of males at each angular bin surrounding the female. Areas of green correspond to the spatial quadrants on either side

of the female along the medial-lateral axis; areas of white correspond to spatial quadrants on either side of the anterior-posterior axis. Forward and sideways velocities are affected following the inactivation of most LC lines; however, the most severe deficits occur when males are along either side of the female along the medial-lateral axis.

d) Relative velocities showing both (1) the mean sideways velocity when the male is on either side of the female's medial-lateral axis (V_{ML}) to the sideways mean velocity when the male is within the female's anterior quadrant (V_A) and (2) the sideways mean velocity when the male is on either side of the female's medial-lateral axis to the mean sideways velocity when the male is within the female's posterior quadrant (V_P). Inactivation of all lines leads to decreased V_{ML}/V_A ratios when compared to controls (n=32/group, $p < 0.001$, One-way ANOVA).

e) Average velocity of males courting intact females (see Figure 3). Plots are as in (c).

f) Average velocity of males courting decapitated females. Plots are as in (c).

g) Males courting decapitated females show a significantly smaller V_{ML}/V_A than controls (n=32/group, $p < 0.05$, One-way ANOVA).

Tap	Ori	Sci	Feature	Description	units
		+	θ_{wings}	Angle between C_{LW} > C_{body} > C_{RW}	rad
		+	θ_{LW}	Angle between C_{LW} > C_{body} > x-axis	rad
		+	θ_{RW}	Angle between C_{RW} > C_{body} > x-axis	rad
		+	A_{LW}	Area of left wing	mm^2
		+	A_{RW}	Area of right wing	mm^2
		+	D_{wing}	Total distance between C_{LW}, C_{body}, C_{RW}	mm
+	+	+	D_{CC}	Male-to-female distance (centroid)	mm
+	+	+	D_{HE}	Male-head to female-ellipse distance	mm
+	+	+	D_{RE}	Male-rear to female-ellipse distance	mm
+	+	+	ΔD	$D_{RE} - D_{HE}$	mm
+	+	+	Θ_{Rel}	Relative heading of male w.r.t. female	rad
+	+	+	$\lvert\Theta_{rel}\rvert$	Absolute value of Θ_{Rel}	rad
+	+	+	v_{Θ}	Angular velocity of male	rad/sec
+	+	+	$\lvert v_{\Theta}\rvert$	Absolute value of v_{Θ}	rad/sec
+	+	+	$\lvert v_{C}\rvert$	Velocity of male centroid	mm/sec
+	+	+	L_{maj}	Maj. axis length of male ellipse	mm
+	+	+	L_{min}	Min. axis length of male ellipse	mm
+	+	+	A	Area of male ellipse	mm^2
+	+	+	Θ	Angle of male ellipse w.r.t. x-axis	rad
+	+	+	D_{CE}	Male-centroid to arena edge distance	mm

Table S1. Features used to generate behavioral classifiers, Related to Figures 1–4.

First and second derivatives were calculated for all features. Additionally, sliding windows, which took into account statistics across multiple videos frames with time frames of up to one second, were also calculated and used in each behavioral classifier. Full details of the features used to classify male courtship behaviors can be found in our code repository on Github (https://github.com/regginold/drosophila-courtship; see Resources Table). Abbreviations are as follows: C_{LW}, centroid of male fly's left wing; C_{RW}, centroid of male fly's right wing; C_{body}, centroid of male fly's body.

Classifier	N_{videos} Scored	N_{frames} Scored		Cross Validation		
		+	-	Acc (%)	FPR (%)	FNR (%)
Tap	8	1137	1431	95.92 ± 1.14	2.21±0.74	1.86±0.74
Ori	8	4243	4412	97.59 ± 1.57	0.62±0.58	1.79±1.55
Sci	8	4277	5248	98.18 ± 1.03	1.37±0.95	0.46±0.18

Table S2. Behavioral classifier cross-validations for control male courtship, Related to Figure 1.

Leave-one-out cross validation was used to determine classifier accuracies, false positive rates (FPRs), and false negative rates (FNRs), as well as standard errors around the mean (± SEM) for each of the behavioral classifiers. Note that independent classifiers were built for each experiment, as described in the methods section.
